# NEURAL STATES ASSOCIATED WITH MOTIVATION AND REWARD MODULATE AGING IN DROSOPHILA

**DOI:** 10.1093/geroni/igad104.0733

**Published:** 2023-12-21

**Authors:** Scott Pletcher

**Affiliations:** University of Michigan Medical School, Ann Arbor, Michigan, United States

## Abstract

Humans, scientists especially, like to believe that we are in control of our behaviors and that logical, rational thought is the driving force behind them. Even for the most conscious of actions, however, there are serious questions about whether this is true. Economists, for example, are well-versed in the notion that consumer behavior is irrational. Why? Psychologists attribute it to the complex evaluation of internal and external cues, which is often need-based but can be strongly influenced by perceived costs and benefits. Molecular neuroscience has gone a step further in recent years, often using simple model organisms, to provide a well-defined framework for dissecting the causes and consequences of motivated behaviors. Homeostatic needs are as influential in this process as are rewarding experiences, and I will discuss evidence that the neurons, neural circuits, and conserved signaling molecules that influence neural states associated with feeding, mating, danger in flies are also important modulators of healthy aging.

